# Analysis of colorectal cancers for human cytomegalovirus presence

**DOI:** 10.1186/1750-9378-4-6

**Published:** 2009-04-16

**Authors:** Cecilia Bender, Donato Zipeto, Carlo Bidoia, Silvia Costantini, Alberto Zamò, Fabio Menestrina, Umberto Bertazzoni

**Affiliations:** 1 Department of Mother and Child, Biology and Genetics, Laboratory of Molecular Biology, University of Verona, Strada Le Grazie, 8. 37134, Verona, Italy; 2 Department of Pathology, University of Verona, Strada Le Grazie, 8. 37134, Verona, Italy

## Abstract

**Background:**

A possible association between human cytomegalovirus (HCMV) infection and colorectal cancer progression has been inferred by the identification in tumour tissues of HCMV antigens and specific viral DNA or RNA sequences. To further investigate the relationship between HCMV and colorectal cancers we developed qualitative and quantitative PCR assay to detect HCMV DNA in 56 formalin-fixed paraffin-embedded (FFPE) tissue samples from patients belonging to 4 different histological phenotypes: adenoma; poorly, moderately and well differentiated adenocarcinomas.

**Results:**

Of the 56 FFPE tested tissue samples, 6 (11%) were positive for HCMV nested PCR amplification, and more precisely 1 (5%) of 20 cases of adenoma and 5 (21%) of 24 cases of moderately differentiated adenocarcinoma. No PCR positivity was obtained in samples from well and poorly differentiated adenocarcinomas.

**Conclusion:**

Our observations suggest that there is no evidence of a direct association between HCMV and colorectal cancer. Moreover, the results obtained are not supportive of a causal role of HCMV in the processes of carcinogenesis and/or progression of colorectal cancer. However, the fact that the virus may present a "hit and run" like-mechanism and HCMV can thus only be detectable at a particular stage of a processing adenocarcinoma, suggests that a significant number of colorectal cancers might have been the subject of HCMV infection that could contribute to trigger the oncogenic differentiation. Our analysis does not exclude the possibility of HCMV infection subsequent viral clearance.

## Background

HCMV is a member of the herpesvirus family and constitutes a major public health problem. HCMV infection is lifelong, and it manifests differently depending on the patient's underlying condition and immunological status. Healthy young people with primary HCMV infection are often asymptomatic but the latent virus can reactivate to cause severe diseases in immunocompromised individuals [1].

Evidence has already been obtained that HCMV gene products are capable of transforming cells *in vitro *and of regulating the expression of important host genes, thus inducing a deregulation of cellular pathways relevant to colon adenocarcinoma pathogenesis [1-6].

Furthermore, the identification of HCMV nucleic acids and antigens in tumour tissues, combined with serological evidence, has led to a suggestion of a possible association between HCMV infection and the progression of certain malignancies. Indeed, HCMV infection has been associated with colon adenocarcinoma by several authors [7-11]. However, other studies have found no evidence of a direct association between HCMV infection and adenocarcinoma malignancy [12-17].

The possible contribution of HCMV in the development and progression of colorectal cancer is thus still controversial. To further investigate whether HCMV infection could be responsible for the induction of colorectal cancers and to obtain a precise assessment of the presence of viral DNA in tissue samples, we have performed qualitative and quantitative PCR analyses for HCMV. We have analysed samples obtained from 58 different patients, including 20 adenomas and 38 adenocarcinomas. In order to study the possible relationship between HCMV infection and cancer differentiation we have compared adenomas with adenocarcinomas belonging to 3 different histological phenotypes (poorly, moderately and well differentiated).

## Results

### Genomic DNA quality control

**Figure 1:**
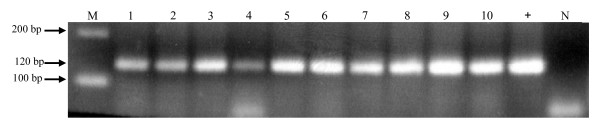
**PCR analysis of *albumin *gene**. DNA extracted from FFPE samples was amplified for *albumin *gene using primers described in Methods. Amplification yielded a band of 120 bp. As positive control (+), human DNA from non-FFPE tissue was used; as negative control (N), PCR master mix without DNA was used. Clinical samples, lanes 1–10. DNA molecular weight marker, M.

DNA was purified from the histological tissues, and its quality was tested using PCR primers targeting for the human *albumin *gene. Fifty six DNA samples (96.5%) of the 58 extracted with xylol/ethanol were positive by *albumin *PCR, and were thus considered suitable for subsequent analysis for the presence of HCMV DNA. A representative PCR analysis of *albumin* gene amplification in FFPE tissue samples is shown in Figure 1. The presence of *albumin *DNA was visualized as a band of 120 bp obtained by using primers specific for *albumin *gene (see Methods). These data indicate that in all examined samples a suitable amplification of *albumin *gene was obtained.

### Nested PCR analysis of HCMV in adenomas and adenocarcinomas

The nested PCR reaction that amplifies a fragment of 180 bp of the HCMV *UL55 *gene (see Methods) was carried out on 56 human DNA samples extracted from adenomas and adenocarcinomas. The results obtained by nested PCR with primers I-1/I-2 are presented in Table 1. The *UL55 *gene was amplified in 1 (5%) of the 20 DNA samples from patients with adenoma, and in 5 (20.8%) of the 24 samples from patients with moderately differentiated adenocarcinoma. No amplification was obtained in any of the 5 cases of well-differentiated adenocarcinoma nor in the 7 cases of poorly differentiated adenocarcinoma.

**Table 1:** Phenotype and PCR results for FFPE samples of colorectal cancer

**clinic phenotype**	**number of samples**	**HCMV PCR positive samples**
**poorly differentiated adenocarcinomas**	7	0
**moderately differentiated adenocarcinomas**	24	5
**well differentiated adenocarcinomas**	5	0
**adenomas**	20	1

**total**	56	6

Since the presence of HCMV DNA was found only in a reduced subset of colorectal cancers, these results suggest that there is no evidence of a direct causal association between HCMV infection and induction of poorly or well-differentiated adenocarcinomas. However, viral DNA was found in approximately 21% of moderately differentiated adenocarcinomas, suggesting that the virus, although not essential to progression towards the transforming phenotype, could conceivably have a role in the initiation of the tumorigenic event.

**Figure 2:**
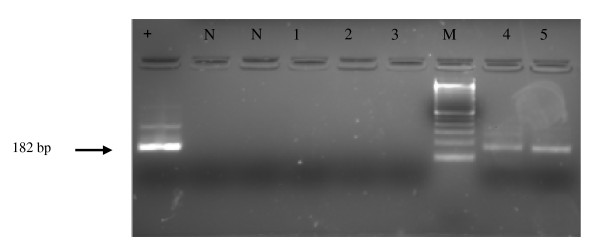
**Nested PCR analysis of viral DNA from representative colorectal cancer samples**. DNA extracted from deparaffinised tissues was amplified with I-1/I-2 primers. Amplification of inner fragment yielded a band of 182 bp. Positive control (+); negative control (N); clinical samples, lanes 1–5; DNA molecular weight marker, M.

In Figure 2 the results obtained for 5 of the 56 clinical samples tested in the second round of PCR are reported; samples 4 and 5 were considered positive since a 182 bp band corresponding to the expected size of viral amplicon was found.

### Quantitative PCR analysis

We developed a real-time PCR quantitative analysis using SYBR^® ^Green I to quantify the viral load in the nested PCR-HCMV positive samples. A standard curve was generated by 10 fold serial dilutions (8 × 10^5 ^– 8 copies) of a plasmid containing a single copy of HCMV *US28 *gene. The regression curve analysis consisting of 5 points tested in triplicate gave a reaction with an efficiency of 100% and a Spearmann's correlation coefficient (R^2^) of 0.99. Quantitative PCR analysis of DNA extracts from FFPE tissues did not allow to obtain reproducible data since the efficiency of PCR was lower (60%, R^2 ^= 0.82) than the 100% standard required. Also the replicate values, from a paraffin-embedded tissue, were discordant (C_t _standard deviation ≥ 1) and the dissociation curve highlighted the presence of non specific amplified products in target samples.

The difficulty in obtaining reliable data from clinical samples is probably linked to the process of paraffin removal that may result in formation of inhibitory substances [18,19] which interfere with the PCR reaction and reduce PCR efficiency [20].

Although this method did not allow quantitation in deparaffinised samples, the real time PCR assay confirmed the qualitative positivity obtained by nested PCR in 2 tested samples (data not shown).

## Discussion

The role of HCMV in the development and progression of colorectal cancers has not yet been clarified. The presence of HCMV antigens and nucleic acids in colorectal cancers has been obtained by means of molecular and virologic studies and a direct relationship between the virus and cancer was suggested [7-11]. However, in other studies, no evidence of a direct association between colorectal cancer and HCMV infection was found [12-17].

More specifically, Harkins et al. [11] have conducted a study on 29 specimens of colorectal polyps and adenocarcinomas from different patients using immunohistochemistry with two different antibodies, and reported the presence of HCMV proteins in about 80% of polyps and 85% of adenocarcinomas, but not in adjacent healthy tissues. In addition, by means of PCR and DNA sequencing, the presence of viral nucleic acids was detected in 6 tumours (5 adenocarcinomas and 1 polyp) that were immunoreactive for HCMV, whereas it was not detected in healthy tissues from 3 of the same 6 patients. These results suggested a possible causative association between HCMV and colorectal cancer. Akintola-Ogunremi et al. [17] examined 23 colorectal hyperplastic polyps, 65 colorectal adenomas and 51 colorectal adenocarcinomas by immunohistochemical analysis with two different antibodies. No nuclear HCMV antigen positivity was detectable in any of the studied cases. In addition, PCR analysis failed to detect viral DNA in 24 selected cases showing non-specific cytoplasmic immunostaining. These results are in stark contrast to those reported by Harkins et al [11], since no evidence of HCMV DNA and proteins in human colorectal adenocarcinomas and their precursor lesions was found.

Knösel et al [16] investigated the presence of HCMV DNA and antigens, by using PCR analysis and immunohistochemistry. Fifty seven primary tumours and 20 metastases of fresh colorectal cancer tissue were tested, including 13 tumour pairs (primary and metastases) from the same patient. Four (7%) of 57 primary tumours were found positive for HCMV DNA by PCR, whereas all metastases were negative. In addition, no specific staining was detected by immunohistochemistry analysis. Although these results confirmed the results of Akintola-Ogunremi et al [17] indicating that there is no direct association between HCMV infection and progression or formation of metastases in colorectal cancer, evidence of HCMV positivity in a small number of colorectal neoplasms was found in the primary tumours.

Given the controversial conclusions of the reported results, we further investigated the issue by establishing a specific qualitative PCR analysis and a quantitative PCR reaction for HCMV viral load determination.

The nested PCR analysis conducted in our laboratory allowed the identification of HCMV sequence in 6 (10.7%) of 56 tissue samples tested, and more precisely in 1 (5%) of 20 cases of adenoma and 5 (20.8%) of 24 cases of moderately differentiated adenocarcinomas. However, there was no evidence of HCMV DNA in any of 5 cases of well-differentiated adenocarcinoma nor in 7 cases of poorly differentiated adenocarcinoma, suggesting the lack of a causative association between virus infection and cancer development in these two groups of adenocarcinomas.

Of interest is the detection of HCMV DNA in 20.8% of moderately differentiated adenocarcinomas, whereas it was undetectable in poorly and well-differentiated carcinomas, similar to the findings of Knösel et al. [16]. It has been reported, in transformed HCMV cells, that the HCMV infection could present a "hit and run" oncogenic-like mechanism [2,21], though viral DNA is undetectable in metastases. It's presence in earlier histological stages may suggest that the presence of HCMV DNA might not be essential to progress toward the transforming phenotype. Following the "hit and run" like mechanism, the virus could mediate cellular transformation through an initial hit, while maintenance of the transformed state is compatible with the loss of viral molecules. Thus, the fact that we have detected viral DNA only in moderately differentiated carcinomas may reflect the inability of HCMV to infect poorly differentiated cancer cells [22].

The quantitative real time PCR analysis was validated on HCMV DNA strains, but could not give reproducible data on DNA extracted from paraffin embedded samples. This could potentially be due to the presence of inhibitors in biopsy samples that can interfere with the PCR. Quantification of HCMV viral load by real-time PCR can yield important information on the timing of HCMV infection. Therefore, validation of the real-time PCR protocol in non-paraffin embedded samples could represent a useful tool for the identification of HCMV in human cancer.

## Conclusion

In conclusion, our observations, together with those of other authors [16,17] suggest that there is no evidence of a direct association between HCMV and colorectal cancer. Moreover, there is no evidence supporting that HCMV can fulfil the necessary criteria for defining a causative role in the process of carcinogenesis and or progression of colorectal cancer. One of these requirements is, in fact, the regular presence and persistence of the nucleic acid of the respective infectious agent in cells of the specific tumour type [23,24].

However, the fact that the virus may present a "hit and run" like-mechanism and that it was found only in a particular stage of a processing adenocarcinomas, provides a useful indication that a certain type of adenocarcinoma have been infected by HCMV and that this could have contributed to trigger the oncogenic differentiation.

## Methods

### Specimen collection

The specimens included in this study consisted of 10% FFPE tissue blocks of colorectal tumours from the archives of the Pathology section of the Department of Pathology, University of Verona, Italy. Samples of 58 biopsies were obtained from different patients of which 20 were adenomas and 38 adenocarcinomas. Of the 38 adenocarcinomas, 5 were well differentiated, 25 moderately differentiated and 8 poorly differentiated.

### Biopsies and histology

Samples were independently reviewed by two pathologists (AZ and FM) and classified according to the WHO classification of tumours of the digestive system. In case of discrepancy, a consensus was reached by reviewing discordant cases at a multi-head microscope. Representative tissue blocks were selected for DNA extraction, selecting blocks with high tumour content.

### Isolation of genomic DNA from paraffin-embedded tissues

Four 3 μm sections of each sample were deparaffinised with xylol/ethanol, collected into a 1.5 ml Eppendorf tube containing 100 μl of lysis solution: 0.1 M NaCl, 0.15 M EDTA pH 8.0, 0.1 M Tris-HCl pH 8.0, 1% SDS, 45.8 mM β-mercaptoethanol and 100 μg Proteinase K. The tubes were incubated at 55°C for 3 hours, followed by Proteinase K heat inactivation. Samples were centrifuged at room temperature at 10.000 g for 5 min. The supernatants were collected and stored at -20°C, prior to the PCR amplification procedure.

### Polymerase chain reaction analysis

Since DNA extracted from paraffin-embedded tissue might be partially degraded during formalin fixation or paraffin embedding [25], DNA quality was tested using the following PCR primers specific for the human *albumin *gene: primer alb fw (5'gctgtcatctcttgtgggctgt 3') and primer alb rv (5'actcatgggagctgctggttc 3'). A total of 56 samples were considered suitable to be tested for the presence of HCMV viral DNA.

The analysis of HCMV in DNA samples was performed by developing a nested PCR reaction with external and internal primers specific for HCMV *UL55 *gene region that encodes for the envelope glycoprotein B. Oligonucleotide primers used to detect HCMV DNA have been described previously [26] and are summarised in Table 2. External primers (E-1/E-2) amplify a 267 bp fragment, while internal primers (I-1/I-2) amplify a 182 bp fragment. All samples were tested in duplicate and samples positive for HCM *UL55 *gene were tested at least in triplicate.

**Table 2:** Primers used for HCMV DNA amplification by nested PCR

**primer**	**sequence**	**amplicon length**	***UL55 *gene position**
E-1	TCCAACACCCACAGTACCCGT	267 bp	655 to 675
E-2	CGGAAACGATGGTGTAGTTCG		902 to 922
I-1	GTCAAGGATCAGTGGCACAGC	182 bp	685 to 705
I-2	GTAGCTGGCATTGCGATTGGT		847 to 867

Primers used for amplification of the HCMV glycoprotein B (*UL55*) gene in nested PCR. External primers amplify a 267 bp fragment, while internal primers amplify a 182 bp fragment

The first-round of PCR amplification was performed in a 20 μl reaction containing 2–5 μl of each genomic DNA sample. As positive controls for PCR reactions 3 different HCMV DNA samples were used: DNA extracted from human fibroblasts cultured *in vitro *and infected with an HCMV strain from a newborn baby with congenital infection and viral DNA of the two laboratory strains AD169 and Towne. Each experiment was conducted using positive and negative controls.

For the first amplification round, after an initial denaturing step at 94°C for 5 min, 35 cycles of denaturation at 94°C for 30 sec, annealing at 58°C for 30 sec, extension at 72°C for 30 sec, were made, followed by a final extension at 72°C for 6 min. The amplified products were subjected to the second round of PCR using the primer pair I-1/I-2 and 1 μl of the first round amplification product. Cycling program was performed as follows: 94°C for 5 min, 1 cycle; 94°C for 30 sec, 59°C for 30 sec, 72°C for 30 sec, 40 cycles; 72°C for 5 min, 1 cycle.

At the end of the amplification 15–20 μl of each product were analysed on 2.5% w/v agarose gels by electrophoresis.

### Real-time PCR

The real-time quantitative PCR assay was performed on an ABI Prism 7000 real time PCR instrument (Applied Biosystems). Primers used for amplification of HCMV *US28 *gene in real-time reaction are summarised in Table 3 and were designed using Primer Express V2.0 software (Applied Biosystems) on the basis of the sequence of *US28 *gene of AD169 laboratory strain (GenBank accession number: NC_001347) and then controlled by aligning sequences with BLAST. The reaction mixture was set using the Real Master Mix 2.5X (Eppendorf AG) containing SYBR^® ^Green I. Primers were used at a final concentration of 300 nM. Each sample was tested in triplicate.

**Table 3:** Primers used for HCMV DNA amplification by real-time quantitative PCR

**primer**	**sequence**	**amplicon length**	**gene position**
US28 S	TCCATCGGCAACTTCTTGGT	65 bp	267671 to 267690
HHV5-US28As	TCGCCGGAGCATTGAATC		267718 to 267735

Oligonucleotide primers used for amplification of the HCMV *US28 *gene in real-time PCR. Primers were designed using Primer Express V2.0 software (Applied Biosystems) on the sequence of *US28 *gene of the AD169 laboratory strain and controlled by aligning sequence with BLAST.

Reactions were performed under the following conditions: 95°C for 5 min, followed by 45 cycles at 95°C for 20 sec and 60°C for 1 min.

Data were collected and analysed by Sequence Detection System Software (Applied Biosystems).

## Competing interests

The authors declare that they have no competing interests.

## Authors' contributions

CB conducted the PCR analysis and optimisation, designed the quantitative PCR study and drafted the paper. DZ participated in the design and coordination of the study, contributed with the manuscript drafting. CB conducted DNA quality analysis. SC did DNA extractions. AZ reviewed the cases and selected blocks for DNA studies. FM enrolled patients, collected samples and reviewed the cases. UB supervised the whole project and gave a significant contribution in drafting the manuscript. All authors read and approved the final manuscript.
